# Microbiome characterization of the sea slugs *Elysia viridis* and *Placida dendritica*: insights into potential roles in kleptoplasty

**DOI:** 10.1186/s12866-025-04573-5

**Published:** 2026-01-02

**Authors:** Patrícia Martins, Paulo Cartaxana, Sónia Cruz

**Affiliations:** Laboratory for Innovation and Sustainability of Marine Biological Resources (ECOMARE), Centre for Environmental and Marine Studies (CESAM), Department of Biology, University of Aveiro, Campus Universitário de Santiago, Aveiro, 3810-193 Portugal

**Keywords:** Bacteria, Carotenoids, High-throughput sequencing, Kleptoplasts, Sacoglossa

## Abstract

**Background:**

Kleptoplasty is the process by which functional chloroplasts from algae food sources are sequestered and retained by a host organism. Some sacoglossan sea slugs display this ability, enabling them to survive extended periods of food shortage, as they can obtain organic compounds from photosynthesis. While research has focused on the mechanisms underlying chloroplast retention and functionality, the contribution of the microbiome to kleptoplasty in these photosynthetic sea slugs is largely unexplored. In this study, we assessed the bacterial communities of *Elysia viridis* and *Placida dendritica*, two photosynthetic sacoglossan species that share the same habitat and macroalga food source, but exhibit distinct abilities to retain chloroplasts.

**Results:**

High-throughput 16S rRNA gene sequencing revealed highly significant differences in bacterial community composition between *E. viridis* and *P. dendritica*. The sea slug *E. viridis* hosted a smaller and more specialized bacterial community, while *P. dendritica* supported a larger, more diverse, and generalist microbiome. *Bacteroidota* and *Actinomycetota* were the most dominant phyla in *E. viridis* (~ 92% of the total sequence reads), while *Pseudomonadota* (former *Proteobacteria*) was the prevalent phylum in *P. dendritica* (~ 66% of the total sequence reads). The dominance analysis revealed that one particularly dominant ZOTU, comprising 224,282 sequence reads (32.5% of total), was found exclusively in *E. viridis* that exhibits long-term retention of functional chloroplasts. According to BLAST search results, this ZOTU was related to the genus *Fulvibacter* (*Flavobacteriaceae* family), known for producing carotenoid-type pigments. These bacteria were not present in the microbiome of *P. dendritica* that shows short-term, non-functional, chloroplast retention.

**Conclusion:**

This study provides novel insights into the microbial communities associated with photosynthetic sea slugs, highlighting significant differences in bacterial composition between *E. viridis* and *P. dendritica*. The identification of carotenoid-producing bacteria in *E. viridis* suggests a possible role in oxidative stress mitigation, and opens new perspectives on the relevance of the microbiome in supporting kleptoplasty. Further research is needed to characterize the functional contributions of bacterial taxa to chloroplast acquisition and maintenance in photosynthetic sea slugs.

**Supplementary Information:**

The online version contains supplementary material available at 10.1186/s12866-025-04573-5.

## Background

Some Sacoglossa sea slugs (Gastropoda, Mollusca) are characterized by their remarkable ability to sequester chloroplasts from algae [1]. The stolen chloroplasts – kleptoplasts – are maintained functionally and capable of harnessing the energy of sunlight, converting it through photosynthesis into usable resources [2]. This capacity, named kleptoplasty, enables these sea slugs to survive extended periods of food shortage, as they can supplement their diet with energy derived from photosynthesis [3, 4]. Studies have revealed that photosynthetic sea slugs may exhibit a wide range of kleptoplast retention times [5, 6]. Some species, like *Placida dendritica*, show non-functional retention, kleptoplasts are rapidly degraded and no photosynthetic activity is detected even immediately after chloroplast acquisition [7]. Others, like *Elysia viridis*, exhibits long-term retention of functional chloroplasts, with photosynthetic activity lasting for some weeks to a few months [8]. Among metazoans, the capacity for long-term maintenance of photosynthetically active kleptoplasts is exclusive to some Sacoglossa, mostly within the genus *Elysia* [9].

Extensive research has been conducted on these photosynthetic sacoglossan sea slugs, particularly focusing on the mechanisms underlying chloroplast sequestration and maintenance. Different sacoglossan species have been found to preferentially feed on and retain functional chloroplasts from specific types of algae, while other species have a more generalist diet, retaining functional chloroplasts from a variety of algae species [6]. The long-term retention species *E. viridis* failed to retain functional chloroplasts when fed on the macroalga *Cladophora rupestris* [10], which indicates a level of selectivity in kleptoplast retention. Therefore, the retention of functional chloroplasts cannot be solely attributed to the alga or sacoglossan individually, depending on a combination of specific characteristics from both species involved [11]. An important and conspicuous factor that remains overlooked in this host-donor relationship is the role of the associated bacteria.

The concept of the “holobiont” has gained increasing recognition, emphasizing the complex and dynamic interactions between a host and its microbiome [12, 13]. Growing awareness of the microbiome’s potential influence on host physiology, metabolism, and adaptation has underscored its fundamental role in shaping the biology of diverse organisms [14]. It has become increasingly evident that many compounds once attributed solely to animal physiology are, in fact, produced by bacterial symbionts [15–17]. Furthermore, symbiotic bacteria contribute to the overall health, metabolism, and ecological interactions of their hosts [18, 19]. Several studies have already demonstrated the importance of microbial communities, and especially bacteria, in maintaining the functionality of algal symbionts in corals [20, 21].

Advances in high-throughput sequencing technologies have revolutionized our ability to understand the complexities of bacterial communities, allowing us to explore their diversity, functional potential, and ecological interactions with unprecedented depth and accuracy [22–24]. However, only few studies have addressed the bacterial communities associated with photosynthetic sacoglossan sea slugs. Devine et al. [25] reported changes in the bacterial diversity associated with two populations of the long-term kleptoplastic sea slug *Elysia chlorotica*, which also differed from its algal prey *Vaucheria litorea*. Two additional studies assessed the bacterial communities of the sacoglossan sea slugs *Elysia rufescens* and *Elysia crispata* [26, 27].

In this study, we characterized the microbial communities of *E. viridis* and *P. dendritica* inhabiting intertidal and shallow coastal waters of the northeastern Atlantic, where they are commonly found on the green macroalga *Codium tomentosum*, their primary food source [28]. Despite sharing the same habitat and algal food source, *E. viridis* can retain functional chloroplasts for up to 12 weeks [29], whereas *P. dendritica* exhibits non-functional chloroplast retention [7]. With this study, we aim to characterize and compare the microbiomes of *E. viridis* and *P. dendritica* using high-throughput sequencing of the 16S rRNA gene. Identification of key bacterial taxa may elucidate their relevance and potential role in the process of kleptoplasty.

## Materials and methods

### Sea slug collection and maintenance

The sacoglossan species *Elysia viridis* (Montagu, 1804) and *Placida dendritica* (Alder & Hancock, 1843) (Supplementary fig. S1), along with their food source, the macroalga *Codium tomentosum* (Stackhouse, 1797), were collected during low tide from the intertidal rocky zone of Tamargueira Beach in Figueira da Foz, Portugal (40° 9’ 58.730” N; 8° 52’ 57.285” W) in mid-September 2024. The animals and algae were maintained under controlled laboratory conditions for three weeks to ensure environmental uniformity prior to sampling. The animals were housed in 150 L recirculating life support systems (LSS) filled with artificial seawater (ASW) at a salinity of 35 PSU and a temperature of 18 °C. The two species, *E. viridis* and *P. dendritica*, were kept in separate maternity nets within the system and regularly fed with *C. tomentosum*. The LSS was operated on a 12 h light:12 h dark photoperiod and equipped with T5 fluorescent lamps, providing a photon scalar irradiance of 60–80 µmol photons m⁻² s⁻¹. Light intensity was measured at the water surface by using a Spherical Micro Quantum Sensor connected to a ULM-500 Universal Light Meter (Walz, Germany). Water changes (approximately 10% of the system’s volume) were performed weekly using freshly prepared ASW, with the same salinity. Additionally, fish maternity nets were replaced once a week.

### Sampling and DNA extraction

After three weeks, the reared animals were collected and washed with autoclaved and filtered ASW to remove transient and loosely attached bacteria. They were then transferred to a disinfected 200 μm pore-sized sieve and thoroughly rinsed with autoclaved and filtered ASW to eliminate debris and excess mucus. The sieve was subsequently placed on tissue paper to remove residual water. Samples were snap-frozen in liquid nitrogen and stored at −20 °C, as DNA extraction was performed within two days of collection.

For *E. viridis*, 10 replicates were prepared, with each replicate consisting of a pool of five individuals. In contrast, for *P. dendritica*, 10 replicates were also used, but each replicate contained a pool of seven individuals due to species-specific differences in body size. Individuals used ranged from 9 to 15 mm in length for *E. viridis* and 4 to 7 mm for *P. dendritica*. Total community DNA (TC) was extracted from whole animal samples of *E. viridis* (10 replicates) and *P. dendritica* samples (10 replicates) using the FastDNA^®^ SPIN Kit (MP biomedicals) following manufacturer’s instructions.

### Next-generation sequencing analysis

The V4 hypervariable region of the 16S rRNA gene amplicons were amplified using PCR primers 515 F (5’-GTGCCAGCMGCCGCGGTAA-3’) and 806R (5’-GGACTACHVHHHTWTCTAAT-3’) [30] with barcode on the forward primer. A 30 cycle PCR assay using the HotStarTaq Plus Master Mix Kit (Qiagen, USA) was performed under the following conditions: 95 °C for 5 min, followed by 30 cycles of 95 °C for 30 s, 53 °C for 40 s and 72 °C for 1 min, and a final elongation step at 72 °C for 10 min. After amplification, PCR products were checked in 2% agarose gel to determine the success of amplification and the relative intensity of bands.

Multiple samples were pooled together in equal proportions based on their molecular weight and DNA concentrations. Pooled samples were purified using calibrated Ampure XP beads, with pooled samples and purified PCR product being used to prepare an Illumina DNA library. Sequencing was performed at MR DNA (www.mrdnalab.com, Shallowater, TX, USA) using Illumina MiSeq platform with paired-end 2 × 250 bp reads, following the manufacturer’s instructions. The average sequencing depth was approximately 20,000 reads per sample. Sequence data were processed using MR DNA analysis pipeline (MR DNA, Shallowater, TX, USA). Briefly, sequences were joined, depleted of barcodes and then sequences < 150 bp or with ambiguous base calls were removed. Sequences were quality filtered using a maximum expected error threshold of 1.0 and dereplicated. After denoising and chimera filtering, zero-radius operational taxonomic units (ZOTUs) were generated. Taxonomic classification was then performed using BLASTn against an internally curated 16S rRNA database (MR DNA version 2024.4) derived from NCBI sequences (https://ftp.ncbi.nlm.nih.gov/blast/db/), as implemented by MR DNA. The database undergoes standard curation and quality-control procedures, including the removal of duplicate, partial, ambiguously annotated, and improperly annotated sequences, and is updated quarterly (versioned by year–quarter). Although the database is proprietary and not publicly available, which limits full reproducibility, MR DNA’s 16S rRNA sequencing and classification pipeline has been widely applied in peer-reviewed microbiome studies, supporting the reliability and consistency of the results obtained.

### Data analyses

An OTU table containing the raw abundance of all ZOTUs of *Elysia viridis* samples (Ev) and *Placida dendritica* samples (Pd) was imported to R version 4.2.3 (http://www.r-project.org/). To assess sequencing effort, rarefaction curves were generated using the rarecurve() function from the vegan package **(**version 2.6–4.6**)** in R.

To determine the distribution of ZOTUs in *E. viridis* and *P. dendritica* samples, a Venn diagram was assembled using the venn() function in the gplots package (version 3.1.3).

Alpha bacterial diversity was assessed by calculating the ZOTUs recorded, Chao1 index, Shannon diversity index and Pielou’s evenness index. All indices were calculated with the diversity() function from vegan package. Differences in bacterial alpha-diversity were assessed using a t-test after confirming normality using Shapiro’s test and homogeneity of variance using the Levene’s test.

All subsequent community composition analyses were performed using relative abundance data, obtained through proportional normalization of read counts within each sample. Variation in ZOTU composition was visualized using Principal Coordinates Analysis (PCoA) based on Bray-Curtis dissimilarity, calculated from relative abundance data with vegdist() function in the vegan package. Ordination plots were generated using the cmdscale() function in R. Differences in the bacterial composition of the two sacoglossan sea slugs were tested using PERMANOVA with the adonis2() function of the vegan package.

For visualization of the representative taxa (Phylum, Class, and Order) in *E. viridis* and *P. dendritica*, stacked bar plots were constructed using the ggplot2 package (version 3.4.4). Prior to plotting, read counts were aggregated at each taxonomic level, normalized to relative abundance (%) within each sample, and taxa contributing less than 1% to the total community were grouped as “Other”.

ZOTUs with more than 3,000 sequence reads were selected to identify the most dominant bacterial communities, and their closest relatives were determined through BLAST search.

The core microbiome of both sacoglossan species was assessed by selecting the ZOTUs that were present simultaneously in all individuals. All DNA sequences generated in this study were submitted to the NCBI SRA: Accession number PRJNA1256232. The R script and input data used for microbiome analysis and figure generation are available via GitHub at: https://github.com/Patriciatmartins/Elysia-viridis-and-Placida-dendritica-Microbiome_.

### Pigment analysis

Pigment extraction and analysis were performed as described by Cruz et al. [31]. Briefly, freeze-dried sea slug and macroalgal samples were extracted for 30 min in 95% cold buffered methanol (2% ammonium acetate), after sonication for 1 min. Extracts were filtered through 0.2 μm PTFE membrane filters before injection into a Prominence-i LC 2030 C HPLC system (Shimadzu, Kyoto, Japan) equipped with a photodiode array detector. Chromatographic separation was carried out using a Supelcosil C18 reverse-phase column (250 × 4.6 mm, 5 μm particle size; Sigma-Aldrich, St. Louis, MO, USA) under a 35 min elution gradient. Pigments were identified based on absorbance spectra and retention times, in comparison with pure pigment standards from DHI (Hørsholm, Denmark).

## Results and discussion

### Overall assessment of bacterial composition

After quality filtering, 689,554 sequence reads were detected and assigned to 194 bacterial ZOTUs. *Elysia viridis* samples displayed 347,778 sequence reads, while *Placida dendritica* presented 341,776 sequence reads (Supplementary table S1).

**Fig. 1:**
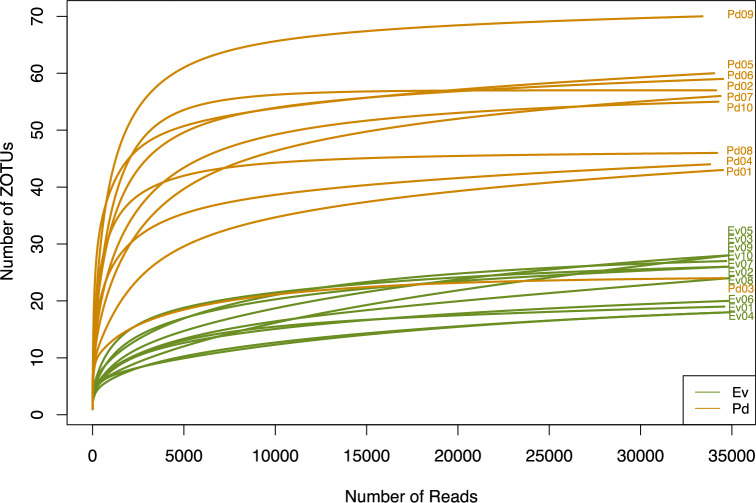
Rarefaction curves illustrating the bacterial diversity and species richness in *Elysia viridis* (Ev) and *Placida dendritica* (Pd) samples

Rarefaction curves illustrate the relationship between sequencing depth (number of sequence reads) and the observed ZOTU richness for each sample (Fig. 1). The asymptotic nature of the curves suggests that sequencing effort was sufficient to capture the bacterial diversity within each sample. Sequence reads were evenly distributed across samples, minimizing biases and supporting robust comparisons between groups. These findings indicate that the dataset provides adequate coverage for assessing bacterial community composition and richness in the studied populations of *E. viridis* and *P. dendritica*.

**Fig. 2:**
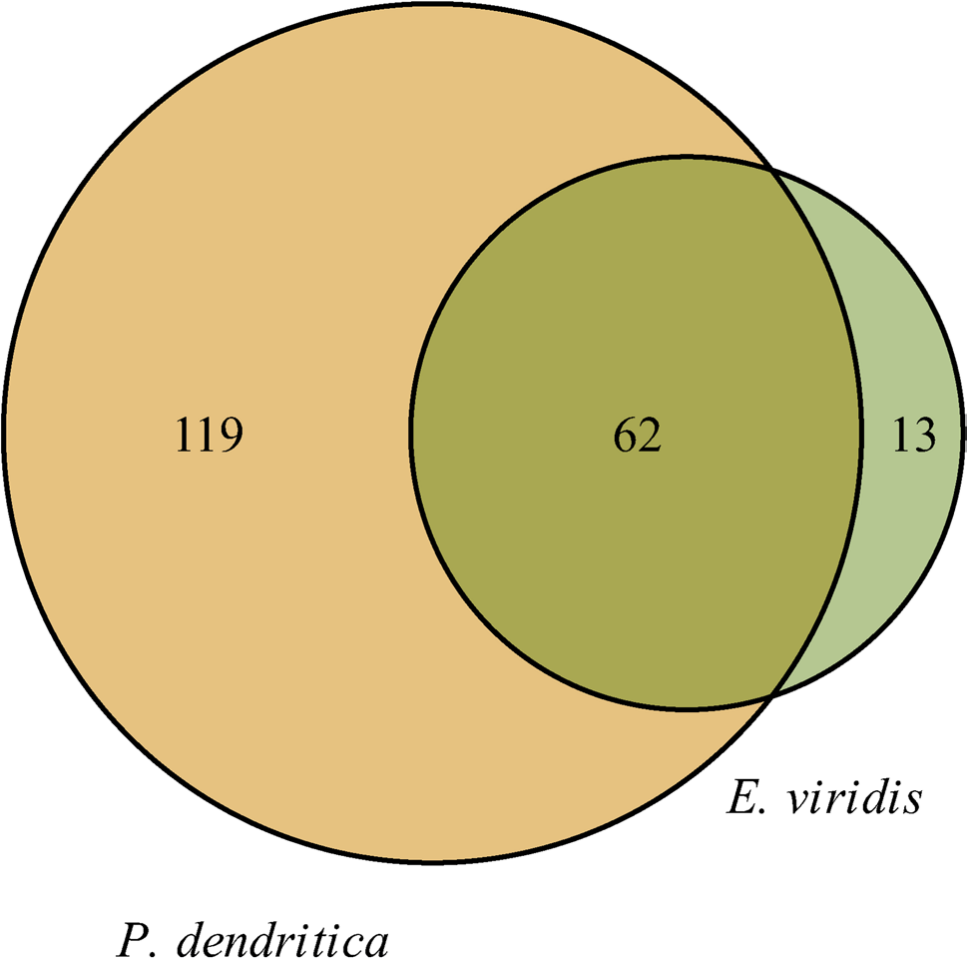
Venn diagram showing the number of shared and unique bacterial ZOTUs in *Elysia viridis* and *Placida dendritica*

The distribution of shared and unique ZOTUs between the samples of *E. viridis* and *P. dendritica* is represented in the Venn diagram (Fig. 2). The sea slug *P. dendritica* contained the highest number of unique ZOTUs, with 119 exclusive to this species, while *E. viridis* samples exhibited only 13 unique ZOTUs. Both species shared a total of 62 ZOTUs, highlighting a core set of microbial taxa present in both organisms. However, if we consider only ZOTUs present in at least 5 replicates, *E. viridis* exhibited only 6 unique ZOTUs, while *P. dendritica* exhibited 13 unique ZOTUs. This reduction in the number of unique ZOTUs could be attributed to transient or low-abundance microbial taxa that were not consistently detected across replicates. These results suggest that, in the Portuguese populations examined, *E. viridis* harbors a smaller yet more specialized bacterial community, whereas *P. dendritica* supports a larger, more diverse, and generalist microbiome.

**Fig. 3:**
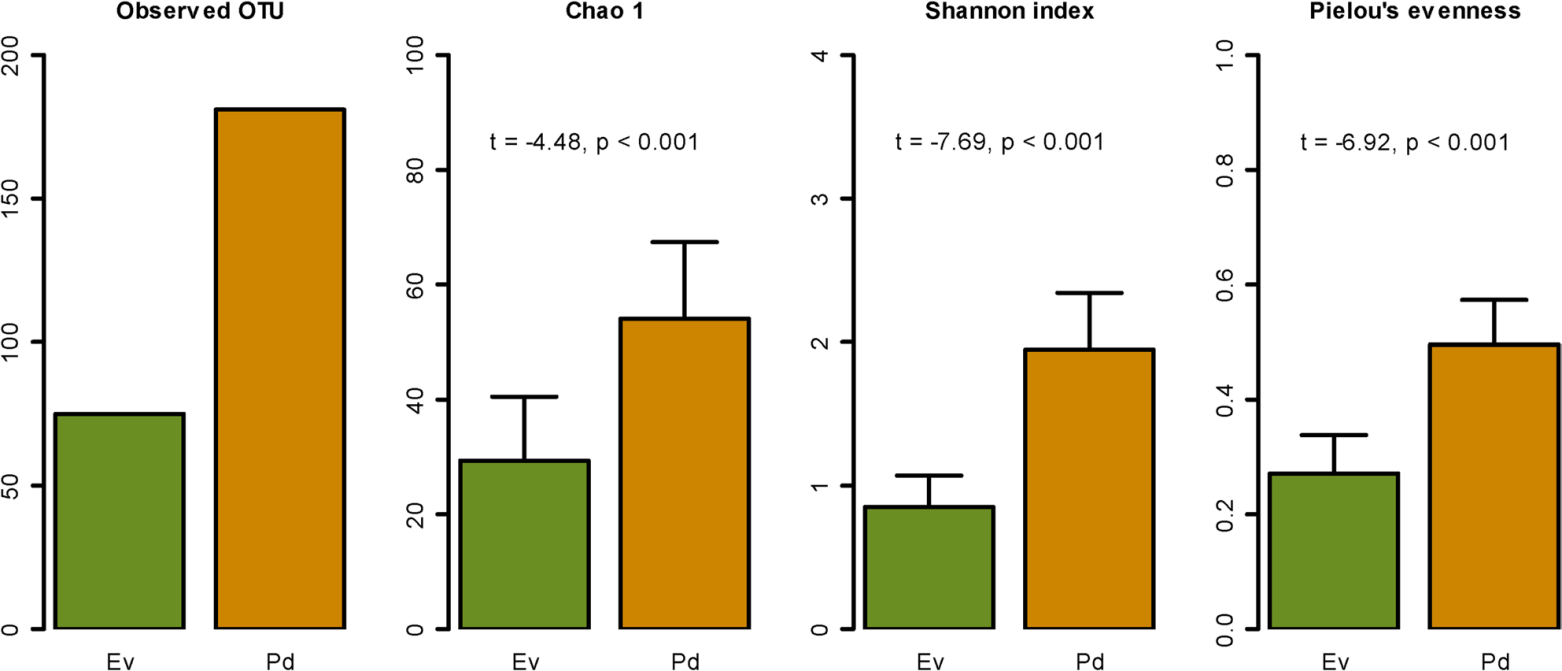
Alpha diversity metrics of bacterial communities associated with *Elysia viridis* (Ev) and *Placida dendritica* (Pd). The graphs display the number of observed ZOTUs, Chao1 richness estimator, Shannon diversity index, and Pielou’s evenness index. Statistical comparisons were performed to assess differences between groups

In terms of bacterial community diversity, results indicate higher alpha diversity in *P. dendritica* samples, as assessed by the Chao1 index, Shannon diversity index, and Pielou’s evenness (Fig. 3). There were statistically significant differences between the bacterial communities associated with *P. dendritica* and *E. viridis*, with *P. dendritica* exhibiting higher values across all diversity indices (Chao1: t = −4.48, *p* < 0.001; Shannon: t = −7.69, *p* < 0.001; and Pielou’s evenness: t = −6.92, *p* < 0.001). These findings suggest that *P. dendritica* microbiome is not only more diverse but also characterized by a well-balanced distribution of bacterial species.

**Fig. 4:**
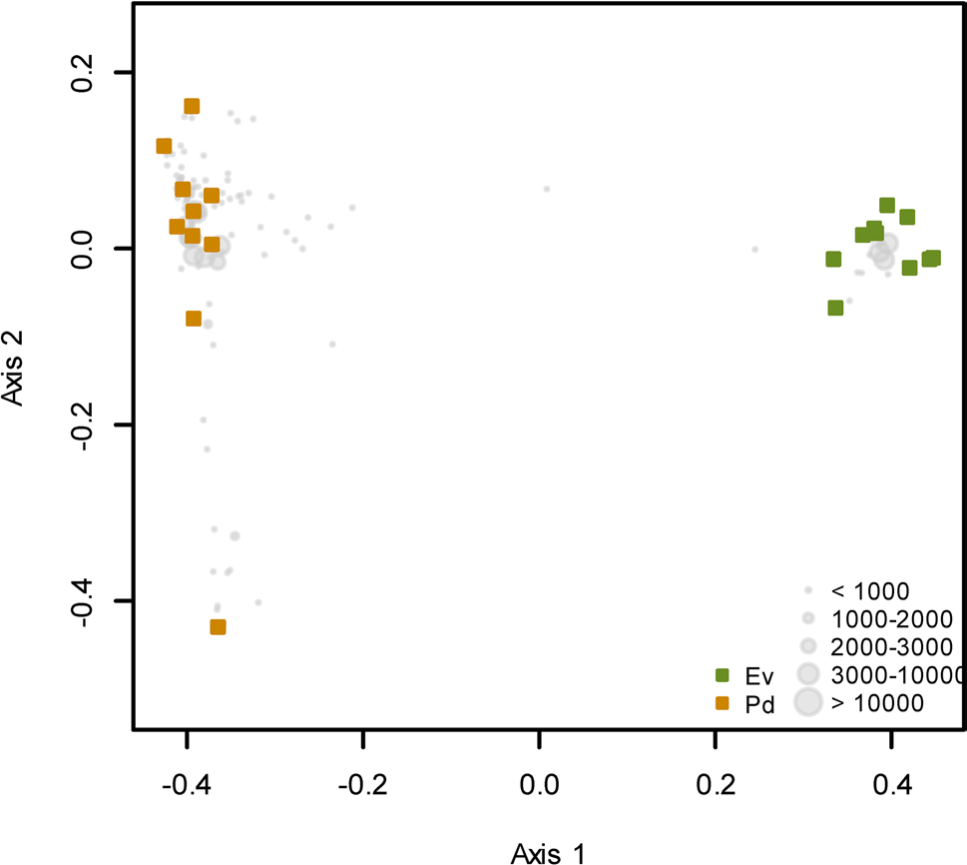
Ordination showing the first two axes of the principal coordinates analysis (PCoA) of the bacterial composition present in *Elysia viridis* (Ev) and *Placida dendritica* (Pd). Grey circles represent ZOTUs, with their size proportional to abundance (number of sequence reads)

The PCoA ordination analysis illustrates a clear separation between two main groups, corresponding to *E. viridis* samples and *P. dendritica* samples (Fig. 4). The primary axis of variation, which explains 66.7% of the variance, highlights the distinct differences in the bacterial communities associated with each species. Statistical analysis revealed highly significant differences in bacterial community composition between *E. viridis* and *P. dendritica* samples (F = 81.058; *p* = 0.001).

### Taxonomic composition of bacterial communities

**Fig. 5:**
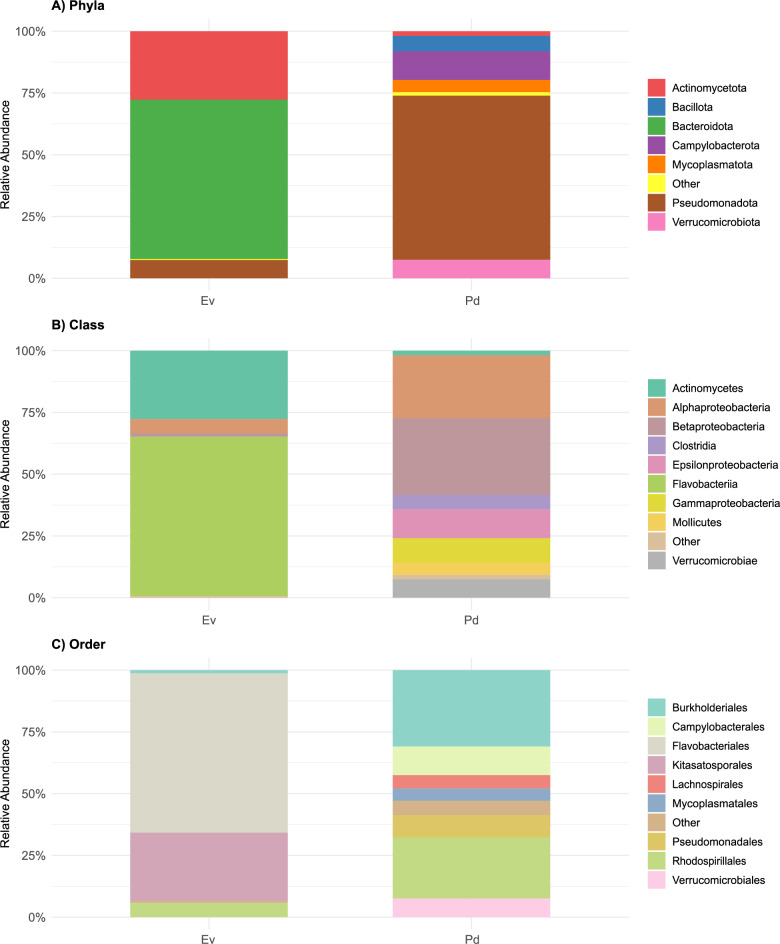
Stacked bar plots showing the relative abundance (%) of representative bacterial groups in *Elysia viridis* (Ev) and *Placida dendritica* (Pd), for phyla (**A**), class (**B**), and order (**C**). Taxa contributing less than 1% to the overall abundance are grouped as “Other”. Bars represent the mean relative abundance (%) based on proportional normalization of read counts within each sample

The overall taxonomic analysis grouped bacterial sequences into fifteen phyla, twenty-nine classes, and fifty-six orders. Fig. 5 highlights the relative abundance of the most representative bacterial groups found in *E. viridis* and *P. dendritica*. *Bacteroidota* and *Actinomycetota* were the most dominant phyla in *E. viridis*, together accounting for approximately 92% of the total sequence reads. In contrast, *P. dendritica* harbored thirteen phyla, with *Pseudomonadota* (former *Proteobacteria*) being the most prevalent, representing 66% of the total sequence reads (Fig. 5A).

*Pseudomonadota*, *Bacteroidota* and *Actinomycetota* are known to dominate the microbial communities in various marine habitats, including in marine invertebrates, such as sea snails, coral and sponges where they contribute to digestion, nutrient cycling, and symbiotic interactions [23, 25, 32].

In a previous study assessing the bacterial communities of the sacoglossan sea slug *E. crispata*, *Spirochaetota* and *Bacteroidota* were reported as the dominant phyla, although *Mycoplasmatota* and *Pseudomonadota* were also detected among other taxa [27]. Similarly, a study investigating the bacterial communities of two wild *E. chlorotica* populations (from near Nova Scotia and Martha’s Vineyard, USA) found a clear dominance of the phylum *Pseudomonadota* [25]. The predominance of the phylum *Pseudomonadota* observed in *P. dendritica*, was also reported in the microbiome of its main food source, the macroalga *C. tomentosum*, in a study examining its microbial community under natural conditions and in response to copper exposure [33].

More than 64% of the bacterial sequences identified in *E. viridis* samples were identified as belonging to the class *Flavobacteriia*, while in *P. dendritica* more than 50% of the bacterial sequences were identified as belonging to the classes *Betaproteobacteria* (31%) and *Alphaproteobacteria* (25%) (Fig. 5B). Previous studies addressing the host-associated microbiome of the sea slug *E. chlorotica*, identified *Betaproteobacteria* and *Alphaproteobacteria* as dominant groups [25]. However, notable differences in the relative abundance of *Betaproteobacteria* were observed between wild populations from Nova Scotia and Martha’s Vineyard, with higher proportions in individuals inhabiting environments that are subject to large salinity fluctuations [25]. These bacterial classes were also among the three most dominant detected in the *C. tomentosum* microbiome, with *Gammaproteobacteria* being the most abundant (57%), followed by *Alphaproteobacteria* (31%) and *Betaproteobacteria*, which accounted for 4.8% [33].

At the order level, 64% of *E. viridis* sequences were grouped under *Flavobacteriales*, with all *Flavobacteriales*-derived sequences being assigned to the *Flavobacteriaceae* family (Fig. 5C). Notably, these dominant families found in *E. viridis* were absent in *P. dendritica*. Members of the *Flavobacteriales* order are recognized for their involvement in the degradation of complex organic matter [34] and play a crucial role in the functioning of marine biofilms [35]. Although *Flavobacteriales* have not been identified as dominant orders in other studies of sea slug microbiomes, they have been observed in both photosynthetic [25] and non-photosynthetic sea slug species [36]. In *P. dendritica*, *Burkholderiales* (30%) and *Rhodospirillales* (24%) were the most abundant bacterial orders, accounting for 54% of the total sequences (Fig. 5C). A previous study by Devine et al. [25] investigating the bacterial communities associated with two populations of *E. chlorotica* also reported that *Betaproteobacteria* in sea slug samples were predominantly represented by the order *Burkholderiales*. These findings suggest that the order-level trends observed in the Portuguese populations of *E. viridis* and *P. dendritica* are consistent with those already reported for other sacoglossan sea slugs, with certain bacterial groups playing a central role in their microbial communities.

### Composition analysis of dominant ZOTUs

The most dominant ZOTUs (≥ 3,000 sequence reads) identified in this study are summarized in Table 1. The dominance analysis revealed four dominant ZOTUs found in *E. viridis*: ZOTU 1 (*Fulvibacter*), ZOTU 2 (unknown *Actinomycetota*), ZOTU 3 (*Ralstonia syzygii*) and ZOTU 8 (*Aestuariispira*) (Table 1). ZOTU 1 was the most abundant ZOTU detected, accounting for 224,282 sequence reads, and found exclusively in *E. viridis* samples (Table 1). According to Blast (http://www.ncbi.nlm.nih.gov/) this ZOTU was related *Fulvibacter tottoriensis* [GenBank accession number (acc.) NR041572] isolated from a marine sediment in Japan [37]. An important characteristic of *Fulvibacter* genus, as observed in many members of the *Flavobacteriaceae* family, is the production of carotenoid-type pigments [37]. Carotenoids are well known for their antioxidant properties, mitigating oxidative damage caused by reactive oxygen species (ROS) [38]. In marine environments, these pigments also play a crucial role in photoprotection from harmful ultraviolet (UV) radiation and intense light exposure [39, 40]. Additionally, carotenoids have been investigated for their role in enhancing stress tolerance in symbiotic and free-living marine organisms [41]. For example, Motone et al. [21] reported the discovery of a zeaxanthin-producing bacterium (belonging to *Flavobacteriaceae* family) with the ability to protect *Symbiodiniaceae* ‒ the algal symbionts of corals ‒ from thermal and light stress, potentially contributing to coral resilience under climate change conditions.

**Table 1:** Taxonomic affiliation of the most abundant ZOTUs in *Elysia viridis* (Ev) and *Placida dendritica* (Pd) (≥ 3,000 sequences) including ZOTU-numbers (ZOTU); number of sequence reads; taxonomic assignment (Phylum, Class and Order); their closest relatives (Blast result); with respective accession number (Accession); sequence identity (Sq ident); and their source. Values represent total (absolute) sequence read counts. All comparative and statistical analyses were performed using relative abundance data normalized by sample read depth

ZOTU	Sequence reads		Sum	Phylum	Class	Order	Blast result	Accession	Sq. ident (%)	Source
	Ev	Pd								
ZOTU1	224,282	0	224,282	Bacteroidota	Flavobacteriia	Flavobacteriales	*Fulvibacter tottoriensis*	NR 114,169	93.94	marine sediment
ZOTU2	96,145	0	96,145	Actinomycetota	Actinomycetes	Kitasatosporales	*Streptomyces* sp.	DQ846821	82.49	European soils
ZOTU3	4,348	97,115	101,463	Pseudomonadota	Betaproteobacteria	Burkholderiales	*Ralstonia insidiosa*	MW960229	100.00	*Ralstonia insidiosa* strain
ZOTU4	0	84,961	84,961	Pseudomonadota	Alphaproteobacteria	unknown	unknown Alphaproteobacteria	HQ675532	81.82	South Atlantic Ocean
ZOTU5	0	39,729	39,729	Campylobacterota	Epsilonproteobacteria	Campylobacterales	*Malaciobacter marinus*	CP032101	93.28	Pacific Ocean
ZOTU6	0	30,705	30,705	Pseudomonadota	Gammaproteobacteria	Pseudomonadales	*Pseudomonas* sp.	MN227209	91.70	*Pseudomonas* sp. strain
ZOTU7	0	25,447	25,447	Verrucomicrobiota	Verrucomicrobiae	Verrucomicrobiales	*Rubritalea marina*	NR_043701	94.86	marine sponge
ZOTU8	19,868	0	19,868	Pseudomonadota	Alphaproteobacteria	Rhodospirillales	*Aestuariispira insulae*	PP053127	89.37	*Aestuariispira insulae* strain
ZOTU9	0	16,573	16,573	Mycoplasmatota	Mollicutes	Mycoplasmatales	*Candidatus* Mycoplasma mahonii	OP995474	95.63	brittle star
ZOTU10	0	13,245	13,245	Pseudomonadota	unknown	unknown	unknown Proteobacteria	LN681284	93.68	marine alga
ZOTU11	0	5,219	5,219	Pseudomonadota	unknown	unknown	unknown Proteobacteria	LN681284	93.28	marine alga
ZOTU12	129	3,312	3,441	Pseudomonadota	Betaproteobacteria	Burkholderiales	*Ralstonia pickettii*	OQ062259	100.00	rice root

Previous studies revealed the presence of an unidentified carotenoid in the sea slug *E. viridis* that was not present in their food source, the macroalgae *C. tomentosum* and *Chaetomorpha* sp [31, 42]. The authors hypothesized that the compound was synthesized by the animal cell using an algal precursor. However, the high abundance and exclusive presence of ZOTU 1 in *E. viridis* suggest a possible bacterial origin for the unidentified carotenoid. To obtain additional insights, we compared the pigment composition of the two sea slug species and the macroalgal food source (Supplementary fig. S2). The pigment profile of *P. dendritica* matched that of *C. tomentosum*, with the presence of chlorophylls *a* and *b*, and the carotenoids siphonaxanthin, all-*trans*-neoxanthin, 9’-*cis*-neoxanthin, violaxanthin, siphonaxanthin dodecenoate, and β,ε-carotene (Supplementary figs. S2B and C). Results confirm that *E. viridis* possesses an additional unidentified carotenoid absorbing in the 400–550 nm range and with an absorption maximum of 460 nm (Supplementary figs. S2A and D). Further investigations are required to elucidate its origin, but it is plausible that a carotenoid produced by the associated bacteria is accumulated by this long-term retention sea slug, helping the animal to cope with oxidative stress generated by the acquired chloroplasts. By mitigating ROS accumulation, this carotenoid could enhance the stability and functionality of kleptoplasts, ultimately benefiting the host organism. While our results highlight a correlation between the dominance of ZOTU 1, a putative carotenoid-producing bacterium, and the long-term retention of functional chloroplasts in *E. viridis*, this association remains speculative and does not establish a direct functional link. Because our analysis is based on 16S rRNA amplicon sequencing, which has limited taxonomic and functional resolution, future studies integrating metagenomic, transcriptomic, and metabolomic approaches, together with experimental manipulation of bacterial communities, will be essential to clarify the specific contributions of the microbiome to chloroplast stability and photoprotection in photosynthetic sea slugs.

ZOTU 2 was classified as an unknown *Actinomycetota* and was detected exclusively in the *E. viridis*. Blast (http://www.ncbi.nlm.nih.gov/) results related this ZOTU to the genus *Streptomyces*, however with a relatively low sequence similarity (< 85%) with organisms from the NCBI database (Table 1). The phylum *Actinomycetota* is one of the most important bacterial groups for the discovery of novel natural products [43]. ZOTU 8 was also dominant in *E. viridis* and was taxonomically assigned to the family *Kiloniellaceae*. BLAST analysis revealed that this ZOTU is closely related to *Aestuariispira insulae*, although with a sequence homology of less than 90% compared to known references (Table 1). Members of the *Kiloniellaceae* family have previously been identified in high abundances in various marine invertebrates, including sponges [44], jellyfish [45] and corals [46], as well as in algae [47].

The most abundant ZOTUs found in *P. dendritica* were ZOTU 3 and 12 (*Ralstonia*), ZOTU 4 (unknown *Alphaproteobacteria*), ZOTU 5 (*Malaciobacter*), ZOTU 6 (*Pseudomonas*), ZOTU 7 (*Rubritalea*), ZOTU 9 (*Mycoplasma*), ZOTU 10 and 11 (unknown *Proteobacteria*) (Table 1). ZOTUs 3 and 12, unlike the other dominant ZOTUs, were detected in both *E. viridis* and *P. dendritica*, however with a significant higher number of sequence reads in *P. dendritica* samples. Blast results related these ZOTU to *Ralstonia insidiosa* and *Ralstonia pickettii* (former *Pseudomonas pickettii*), respectively (Table 1). While both species are primarily recognized as opportunistic pathogens associated with plant and human diseases [48, 49], they are also known for their capacity to form robust biofilms [50]. Previous studies have demonstrated that members of the genus *Ralstonia* produce signaling molecules involved in quorum sensing and biofilm formation [51], although the extent and mechanisms may vary among species. Additionally, they exhibit exceptional metabolic versatility, enabling them to use a wide range of carbon sources, including aromatic compounds [52]. This metabolic flexibility allows the bacterium to thrive in a variety of environmental conditions, further contributing to its persistence in diverse habitats.

According to BLAST analysis, ZOTU 5 was identified as *Malaciobacter marinus* (formerly *Arcobacter marinus*) (Table 1). Although the ecological role of *Malaciobacter* in the marine environment remains unclear, it has been reported as a prevalent and abundant bacterial species in the microbiomes of oysters (*Tiostrea chilensis*) and corals (*Acropora* species) [53, 54]. Additionally, members of this genus have been detected in the skin and gill mucus of various fish species [55].

Blast results related ZOTU 6 to the genus *Pseudomonas*, a group of bacteria that are ubiquitous in marine environments. However, the sequence similarity was relatively low (91%) when compared to organisms in the NCBI database (Table 1). *Pseudomonadaceae* family plays a crucial role in the nitrogen and carbon cycles by fixing nitrogen, decomposing organic carbon, and converting nitrogenous compounds, thereby contributing to ecosystem balance [56, 57]. Additionally, several members of this family are known for producing antimicrobial compounds, including antibiotics and bacteriocins. Some *Pseudomonas* species also establish symbiotic relationships with marine organisms, such as fish and corals, supporting host health by promoting nutrient cycling and acting as biocontrol agents against harmful pathogens [58–60]. This family has previously been identified in the microbiome of sea slugs [35] including photosynthetic species [25].

BLAST analysis identified ZOTU 7 as *Rubritalea marina*, which was exclusively detected in *P. dendritica*, accounting for 25,447 sequence reads (Table 1). This species was originally isolated from the Mediterranean sponge *Axinella polypoides* [61]. The prevalence of *Rubritalea* species in marine sponges suggests their potential role in symbiotic interactions with marine organisms. Members of this genus are known for their ability to produce carotenoids and degrade organic matter, contributing to both carbon and nitrogen cycling [62].

ZOTU 9, detected exclusively in *P. dendritica*, was assigned to *Candidatus* Mycoplasma mahonii based on a BLAST search. Although generally regarded as parasitic, *Mycoplasma* species are frequently found in symbiotic associations with marine organisms, including fish and corals [63, 64]. They are integral members of the normal microbiota of these hosts, colonizing mucosal surfaces and the digestive tract [64, 65]. Their versatility makes them well-suited for host-associated niches such as fish gills, intestines, and coral mucus layers. As facultative anaerobes or microaerophiles, *Mycoplasma* species can thrive in oxygen-limited conditions, further supporting their adaptation to these environments [66]. Members of the genus *Mycoplasma* have been already detected in photosynthetic sea slugs, including *Elysia crispata* [67] and *Elysia rufescens* [26]. Although the microbiomes of *P. dendritica*, *E. crispata*, and *E. rufescens* share the presence of specific genera such as *Pseudomonas* and *Mycoplasma*, their overall bacterial communities exhibit significant differences at the genus level. This is not unexpected, considering the differences in their environments, feeding strategies, and dietary preferences.

It is important to highlight that, among all the most abundant ZOTUs detected in the studied *E. viridis* and *P. dendritica* populations, only two (ZOTU 3 and 12) showed 100% sequence homology in the BLAST search. Six ZOTUs exhibited homology between 93% and 95% (ZOTU 1, 5, 7, 9, 10 and 11), two ZOTUs demonstrated homology ranging from 88% to 92% (ZOTU 6 and 8), while the remaining two displayed homology below 85% (ZOTU 2 and 4) in comparison to published sequences (Table 1). These findings suggest that the taxonomic assignment of several abundant ZOTUs remains uncertain at the species level, highlighting potential novel or highly divergent bacterial lineages associated with *E. viridis* and *P. dendritica*. The marine environment represents a vast and largely unexplored reservoir of biodiversity, with significant potential for the discovery of novel species. In recent years, numerous previously unidentified taxa have been described, including bacterial species associated with sea slugs, highlighting the complexity and ecological significance of host-associated microbiomes [15, 68]. The relatively low sequence homology observed for several dominant ZOTUs in this study suggests the potential presence of yet-undiscovered bacterial species, underscoring the uniqueness of these underexplored microbial communities. Future research should focus on the detailed characterization of these specific bacterial taxa to better understand their functional roles and evolutionary significance for photosynthetic sea slugs, including their putative involvement in kleptoplast establishment and maintenance. At the same time, it should be acknowledged that the present study, conducted without temporal replication and limited to a single sampling location, represents a snapshot from the Portuguese coast that may partly reflect local environmental influences. Expanding spatial and temporal sampling will be necessary to evaluate the consistency of these microbial associations across different habitats and populations.

### Core microbiome characterization

The core microbiome is defined as the set of ZOTUs that are consistently present across all replicates. In *E. viridis*, the core microbiome comprised eight ZOTUs out of the 75 detected. In contrast, *P. dendritica* exhibited a core microbiome comprising nine ZOTUs out of the 181 identified (Supplementary table S2). The core microbiome of *E. viridis* was composed of five phyla, seven classes, seven orders, and seven families, while that of *P. dendritica* comprised six phyla, eight classes, eight orders, and eight families. *Bacteroidota* was the dominant phylum in the core microbiome of *E. viridis*, accounting for approximately 64% of total sequence reads. In contrast, *Pseudomonadota* dominated the core microbiome of *P. dendritica*, representing about 68%. At the family level, *Flavobacteriaceae* (64%) was the most abundant in *E. viridis*, whereas *P. dendritica* was dominated by *Burkholderiaceae* (26%) and *Rhodospirillaceae* (31%).

The taxonomic composition of the core microbiome of *E. viridis* and *P. dendritica* mirrors that of the most dominant ZOTUs identified in each species, because all the dominant ZOTUs identified in *E. viridis* and *P. dendritica* are part of the species core microbiome. All nine ZOTUs constituting the core microbiome of *P. dendritica* were identified as the dominant ones. In contrast, among the eight core ZOTUs of *E. viridis*, four were identified as dominant (ZOTU 1, 2, 3, and 8), whereas ZOTU 12, although dominant in *P. dendritica*, was not dominant in *E. viridis*. Additionally, three other ZOTUs (ZOTU 21, 22, and 24) were not dominant in either of the sea slug species. Those three ZOTUs exhibited a lower number of reads compared to the dominant taxa (< 3,000 sequence reads), with a total of only 581, 618, and 585 sequence reads, respectively. According to BLAST analysis, they were identified as *Candidatus* Mycoplasma mahonii (94.05% sequence homology), *Reinekea* sp. (87.5% sequence homology), and *Gimesia algae* (76.54% sequence homology) (Supplementary table S2).

## Conclusions

This study provides novel insights into the bacterial communities associated with two sacoglossan sea slugs, *E. viridis* and *P. dendritica*, using a culture-independent approach based on 16S rRNA gene sequencing. Our findings reveal that *E. viridis* harbors a distinct and less diverse microbiome, characterized by the dominance of a few highly abundant ZOTUs, such as ZOTU 1 (*Fulvibacter*). Members of the genus *Fulvibacter* and the *Flavobacteriaceae* family are known for their carotenoid production, which may help mitigate oxidative stress associated with photosynthesis, potentially supporting the functionality of kleptoplasts in *E. viridis*. In contrast, *P. dendritica* supports a broader and more diverse bacterial community, likely providing functional redundancy and metabolic adaptability. The microbial assemblage in *P. dendritica* may support its heterotrophic lifestyle by facilitating nutrient acquisition and contributing to essential metabolic functions and defense. It is important to note that this study is based on evidence derived solely from 16S rRNA sequencing, which limits functional inference. Experimental and multi-omics approaches will be required to verify whether the bacterial taxa identified, particularly *Fulvibacter*, directly contribute to carotenoid biosynthesis and oxidative stress regulation in *E. viridis.*

Further research should characterize the identified bacterial taxa and elucidate their specific functional roles within sacoglossan sea slugs. Additionally, the low sequence homology observed between several ZOTUs and known database sequences suggests the presence of previously unidentified species, highlighting the uniqueness and ecological significance of these underexplored microbial communities.

Finally, it should be acknowledged that this study focused on populations of *E. viridis* and *P. dendritica* from the Portuguese coast, and therefore, the results should be interpreted within this geographic context. The core microbiome of these species may vary in other coastal regions due to environmental differences and local adaptations, which could shape host–microbe interactions.

## Supplementary Information

Supplementary Material 1.

Supplementary Material 2.

Supplementary Material 3.

## Data Availability

Raw sequencing data supporting the findings of this study have been deposited in the NCBI Sequence Read Archive (SRA) under the accession number PRJNA1256232. The data are publicly available and can be accessed at: [https://www.ncbi.nlm.nih.gov/bioproject/PRJNA1256232/](https://www.ncbi.nlm.nih.gov/bioproject/PRJNA1256232).
