# Supplementary material for: Microbiome characterization of the sea slugs Elysia viridis and Placida dendritica: insights into potential roles in kleptoplasty
Source: BMC Microbiol. 2026 Jan 2;26:19. doi: 10.1186/s12866-025-04573-5 (PMC12781374; doi:10.1186/s12866-025-04573-5)
Supplement: Supplementary file 1 — Supplementary Material 1. [file 12866_2025_4573_MOESM1_ESM.pdf]

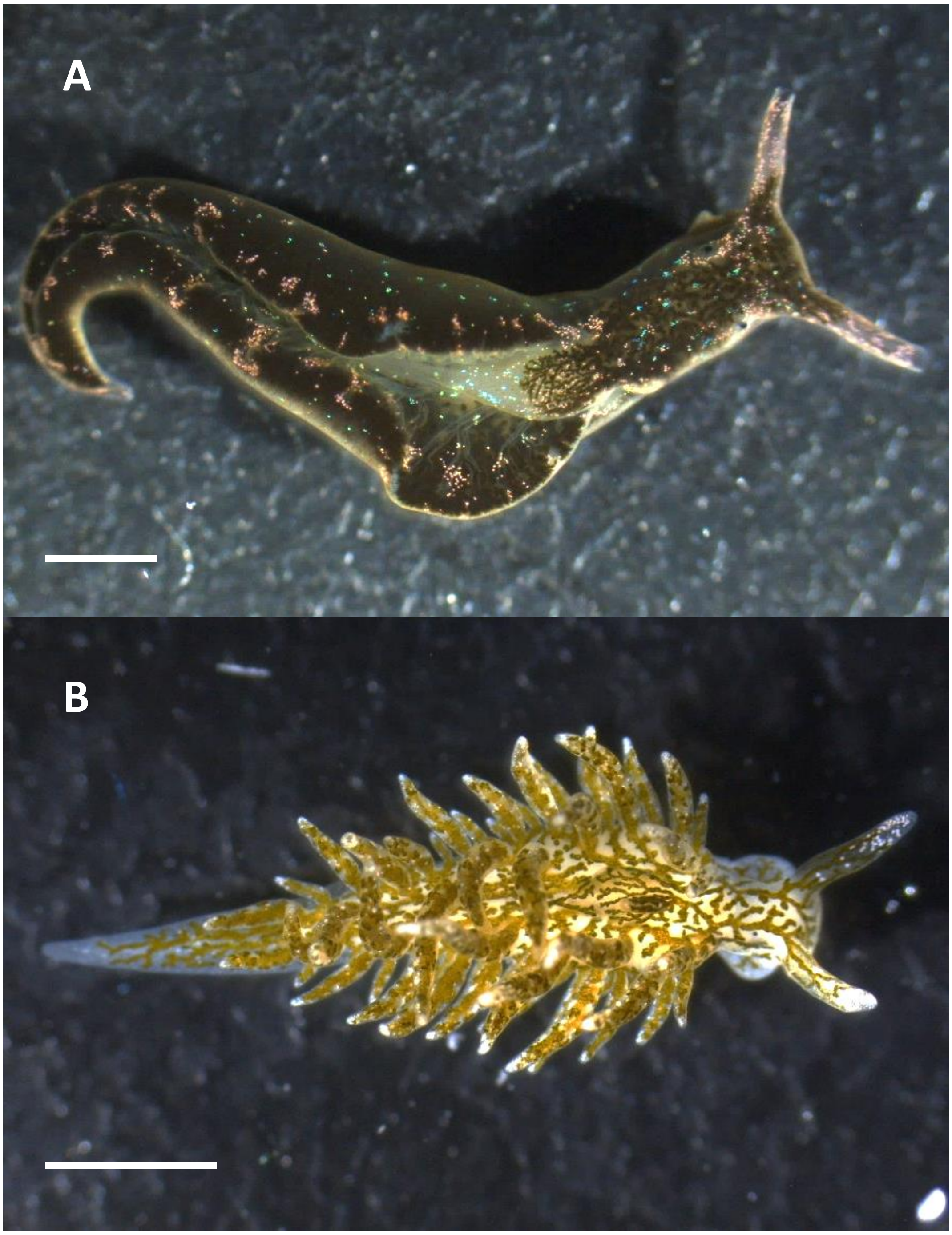

Supplementary Figure S1. The sacoglossan sea slugs *Elysia viridis* and *Placida dendritica*. The sea slug *E. viridis* (**A**) displays long-term maintenance of functional chloroplasts, while *P. dendritica* (**B**) shows short-term, non-functional, chloroplast retention. Both sea slug species share habitat and the macroalga *Codium tomentosum* as source of the stolen chloroplasts. Photos obtained using a digital microscope (DMS-300, Leica Microsystems). Scale bars represent 1 mm.
