# Supplementary material for: Microbiome characterization of the sea slugs Elysia viridis and Placida dendritica: insights into potential roles in kleptoplasty
Source: BMC Microbiol. 2026 Jan 2;26:19. doi: 10.1186/s12866-025-04573-5 (PMC12781374; doi:10.1186/s12866-025-04573-5)
Supplement: Supplementary file 2 — Supplementary Material 2. [file 12866_2025_4573_MOESM2_ESM.pdf]

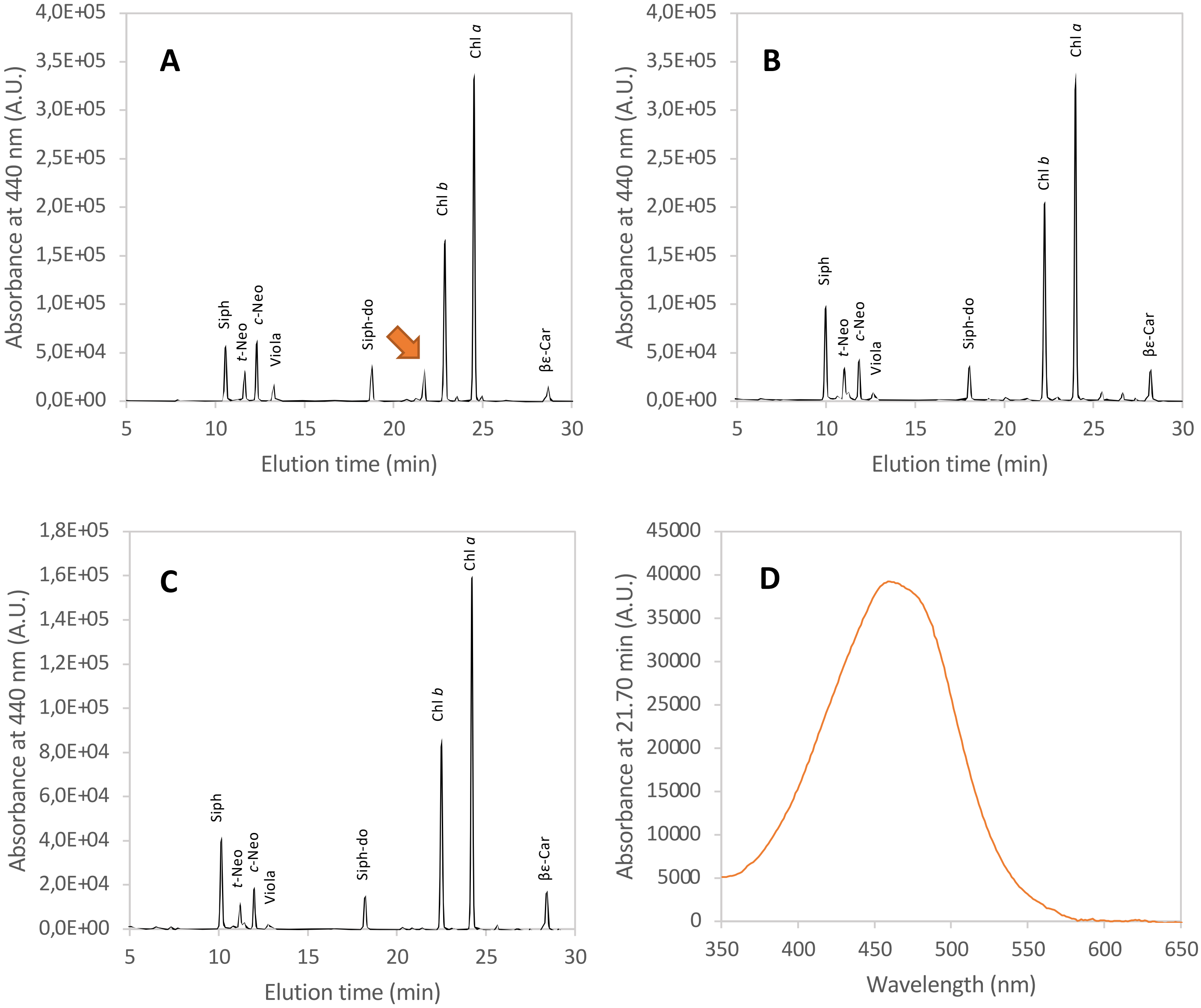

Supplementary Figure S2. Pigment profiles of the sea slugs *Elysia viridis* and *Placida dendritica,* and the macroalga *Codium tomentosum,* food and kleptoplast source of the two sea slugs. HPLC chromatograms at 440 nm of *E. viridis* (**A**), *P. dendritica* (**B**), and *C. tomentosum* (**C**). The sea slug *E. viridis* shows an additional unidentified carotenoid that was not present in *P. dendritica* or *C. tomentosum*. The unidentified carotenoid (indicated in **A** by an arrow) eluted at 22 min and showed a maximum absorption at 460 nm (**D**). Identified pigments: siphonaxanthin (Siph), all-*trans*-neoxanthin (*t*-Neo), 9'-*cis*-neoxanthin (*c*-Neo), violaxanthin (Viola), siphonaxanthin dodecenoate (Siph-do), chlorophyll *b* (Chl *b*), chlorophyll *a* (Chl *a*), and β,ε-carotene (βε-Car).
